# Refractory Clostridioides difficile Colitis in a Patient With Hidradenitis Suppurativa: Consequences of Long-Term Clindamycin and Multi-agent Anti-TNF Therapy

**DOI:** 10.7759/cureus.104900

**Published:** 2026-03-09

**Authors:** Cameron I Blanchard, Drew K Knight, Alande Brezault

**Affiliations:** 1 School of Medicine, St. George's University, St. George's, GRD; 2 Department of Internal Medicine, I and B Medical Associates, Miami, USA; 3 Department of Internal Medicine, Jackson Memorial Hospital, Miami, USA

**Keywords:** anti-tnf therapy, clindamycin, clostridioides difficile infection, hidradenitis suppurativa, immunosuppression, refractory cdi, vancomycin

## Abstract

*Clostridioides difficile* infection (CDI) is a significant cause of morbidity and mortality worldwide, and its treatment is often challenging in patients with complex medication regimens. We report a case of refractory CDI in a 70-year-old woman with Hurley stage II hidradenitis suppurativa (HS) involving the right axilla and perianal region. Her HS was managed with chronic antibiotic use (clindamycin) and anti-tumor necrosis factor (TNF) biologics, initially infliximab (5 mg/kg every eight weeks) followed by adalimumab (40 mg every two weeks) due to inadequate response to infliximab. Despite multiple hospitalizations and treatment with vancomycin and fidaxomicin, the patient experienced recurrent CDI with systemic manifestations including fever, tachycardia, and altered mental status. This case illustrates how chronic antibiotic-induced dysbiosis combined with immunosuppressive therapy can create treatment-resistant CDI requiring multidisciplinary management beyond standard antibiotic protocols.

## Introduction

*Clostridioides difficile* (*C. difficile*) is a gram-positive, spore-forming, anaerobic bacterium that colonizes the gastrointestinal tract of many healthy people. While present in the normal gut flora of many individuals, disruption of the microbiome, particularly from antibiotic therapy, can lead to overgrowth and symptomatic infection [1]. It is one of the most common hospital-acquired infections, and its frequency and severity have been increasing worldwide. Historically, the antibiotics of choice for *C. difficile* infection (CDI) are vancomycin and fidaxomicin. Standard CDI treatment has recently improved with fidaxomicin, demonstrating superior clinical cure rates to vancomycin with significantly lower recurrence rates, yet certain populations remain resistant to this conventional therapy. A recent retrospective study showed fidaxomicin had a 63% reduction in the composite risk of clinical failure, 30-day relapse, or CDI-related death compared to vancomycin [2].

Hidradenitis suppurativa (HS) is a chronic inflammatory skin condition often requiring long-term antibiotic and biologic agent suppression [3]. While necessary for disease control, this treatment approach significantly increases susceptibility to opportunistic infections. Chronic antibiotic therapy for HS significantly increases CDI risk, with advanced patient age, multiple antibiotic classes, and frequent hospitalizations serving as additional risk factors [4].

Specifically, clindamycin, though effective for HS, significantly disrupts protective anaerobic gut flora. A meta-analysis of the Food and Drug Administration (FDA) Adverse Event Reporting System found lincosamides (clindamycin) had the highest CDI reporting odds ratio among antibiotic classes evaluated, at 46.95 (95% CI 39.49-55.82) [5]. Additionally, tumor necrosis factor (TNF)-alpha inhibitors used in HS management may further compromise intestinal immune defenses. TNF-alpha promotes neutrophil recruitment and maintains epithelial barrier integrity; its inhibition potentially impairs the gut's ability to respond to *C. difficile* toxins [6]. This report describes a patient whose dermatologic treatment regimen created conditions highly resistant to standard CDI therapy, requiring consideration of alternative management strategies.

## Case presentation

A 70-year-old woman with type 2 diabetes mellitus (T2DM), stage 3a chronic kidney disease (CKD), Hurley stage II HS, and hypertension presented to the clinic with refractory CDI. She had been receiving long-term clindamycin and biologic therapy (initially infliximab, later switched to adalimumab 40 mg every two weeks) for the management of HS with perianal involvement. Her home medications included duloxetine 30 mg daily, losartan-hydrochlorothiazide 50-12.5 mg daily, furosemide 40 mg daily, atorvastatin 20 mg daily, and insulin glargine 25 units at bedtime. 

At a primary care visit, the patient reported fatigue and episodic shortness of breath with exertion. Laboratory evaluation on day 1 revealed several pertinent abnormalities. Complete blood count (CBC) indicated a microcytic anemia with decreased hemoglobin, hematocrit, and mean corpuscular volume (MCV). This was consistent with anemia of chronic disease in the setting of her underlying comorbidities. A comprehensive metabolic panel (CMP) revealed an elevated creatinine with a decreased estimated glomerular filtration rate (eGFR), reflecting worsening of her known stage 3a CKD compared to baseline values earlier in the year. Potassium was borderline elevated, and bicarbonate was decreased, indicating early metabolic acidosis. Albumin was decreased, and total protein was elevated, raising concern for an inflammatory or dysproteinemic process. Concurrent serum protein electrophoresis demonstrated elevated beta-2 and gamma globulin fractions with decreased albumin, showing an overall pattern consistent with an acute phase reaction and active systemic inflammation (Table 1). Skin examination demonstrated fibrotic scarring and indurated plaques in the right axilla and perianal region, consistent with Hurley stage II HS, without active drainage or discharge. 

**Table 1:** Laboratory investigations across clinical course H = high (above reference range); L = low (below reference range); NP = not performed; CMP = comprehensive metabolic panel; CBC = complete blood count; SPEP = serum protein electrophoresis; eGFR = estimated glomerular filtration rate; BUN = blood urea nitrogen; Cr = creatinine; MCV = mean corpuscular volume; WBC = white blood cell; EIA = enzyme immunoassay *SPEP collected on day 1 only; pattern consistent with acute phase reaction; elevation of beta-2 globulin suggestive of acute inflammation. **C. difficile stool assay toxin A & B testing was performed across five clinical encounters: during the first hospitalization (day 4-8), the second hospitalization (day 35-41), the third hospitalization (day 79-83), and two outpatient collections on day 109 and day 169; exact inpatient collection dates were not available from available records and results were confirmed per discharge documentation.

Laboratory panel	Parameters	Reference range	Baseline	Day 1	Day 70	Day 109	Day 169
CMP	BUN	7–20 mg/dL	26 (H)	26 (H)	42 (H)	NP	34 (H)
	Creatinine	0.50–1.10 mg/dL	1.17 (H)	1.37 (H)	1.67 (H)	NP	1.21 (H)
	eGFR	≥60 mL/min/1.73m²	51 (L)	42 (L)	33 (L)	NP	48 (L)
	BUN/Cr ratio	10–20	22 (H)	19	25 (H)	NP	28 (H)
	Sodium	136–145 mmol/L	141	136	134 (L)	NP	139
	Potassium	3.5–5.1 mmol/L	4.4	4.7	5.3 (H)	NP	5.6 (H)
	Chloride	98–107 mmol/L	107	104	108 (H)	NP	112 (H)
	Carbon dioxide, total	22–29 mmol/L	21 (L)	21 (L)	17 (L)	NP	19 (L)
	Albumin	3.5–5.0 g/dL	3.4 (L)	3.0 (L)	3.3 (L)	NP	NP
	Total protein	6.3–8.2 g/dL	9.0 (H)	9.6 (H)	9.8 (H)	NP	NP
CBC	WBC	3.84–10.8 × 10³/µL	9.5	8.7	NP	NP	7.7
	Hemoglobin	12.0–16.0 g/dL	9.1 (L)	8.9 (L)	NP	NP	9.0 (L)
	Hematocrit	37.0–47.0%	29.5 (L)	30.3 (L)	NP	NP	30.2 (L)
	MCV	80.0–100.0 fL	84	78.9 (L)	NP	NP	80
SPEP*	Total protein	6.3–8.2 g/dL	NP	9.8 (H)	NP	NP	NP
	Albumin	3.5–5.0 g/dL	NP	3.1 (L)	NP	NP	NP
	Alpha-1 globulin	0.1–0.4 g/dL	NP	0.4	NP	NP	NP
	Alpha-2 globulin	0.4–1.0 g/dL	NP	0.8	NP	NP	NP
	Beta-1 globulin	0.2–0.6 g/dL	NP	0.6	NP	NP	NP
	Beta-2 globulin	0.4–0.9 g/dL	NP	1.2 (H)	NP	NP	NP
	Gamma globulin	0.7–1.7 g/dL	NP	3.8 (H)	NP	NP	NP
*C. difficile* stool assay**	Toxin A & B (EIA)	NP	NP	NP	NP	Positive	Negative

The patient presented to the hospital at an outside facility on day 4 with persistent diarrhea, tachycardia, abdominal pain, and fever. Patient remained hospitalized until day 8 with a formal diagnosis of CDI. Inpatient treatment consisted of intravenous fluids, oral vancomycin, and discontinuation of her clindamycin regimen. 

The patient was hospitalized at an outside facility from day 4 to day 8, after presenting with persistent diarrhea, tachycardia, abdominal pain, and fever. During admission, a formal diagnosis of CDI was made, and treated with oral vancomycin and discontinuation of the patient's clindamycin regimen. Despite treatment, the patient developed recurrent symptoms requiring a second hospitalization from day 35 to day 41, and was diagnosed with recurrent CDI. Treatment included a 10-day course of fidaxomicin 200 mg twice daily and outpatient follow-up with an infectious disease specialist. At follow-up, the specialist confirmed completion of her fidaxomicin regimen, no signs or symptoms of active infection, and continued observation off antibiotics with instructions to contact the office should any new symptoms arise.

Laboratory evaluation on day 70 revealed acute kidney injury superimposed on her known CKD, metabolic acidosis, and hyponatremia (Table 1). Refractory to guideline-recommended therapy, the patient required a third hospitalization from day 79 to day 83, with alarming symptoms of altered mental status, weakness, hypotension, and fever of 101.4°F. A diagnosis of recurrent CDI complicated by further renal deterioration was confirmed, and the patient received fidaxomicin for three days during admission with a seven-day supply to complete therapy upon discharge. The patient followed up with her primary care physician on Day 86, appearing alert and pleasant with no acute distress, mild left lower quadrant tenderness, clear lungs, and regular cardiac rhythm. Vital signs were notable for a heart rate of 99 beats per minute, blood pressure of 137/74 mmHg, and temperature of 98.1°F, with oxygen saturation remaining stable. Due to continued abdominal discomfort, the patient was referred to gastroenterology for further evaluation of her refractory disease course. Gastroenterology consultation on day 98 revealed the patient’s persistent gastrointestinal symptoms, including bloating, intermittent lower abdominal discomfort, heartburn, and frequent diarrhea, as well as associated systemic symptoms of back pain, dizziness, and fatigue. The gastroenterologist assessed the patient as responding to recent treatment and recommended observation.

After completion of a second round of fidaxomicin, the patient returned to her primary care physician on day 107. She reported new concerns, including significant acid reflux, for which her gastroenterologists advised discontinuing omeprazole and the use of over-the-counter Gaviscon with famotidine as needed. The patient further expressed significant anxiety, noting a recent change in stool color to yellow with the characteristic odor associated with her previous CDI episodes appearing a few days later. Physical examination revealed an alert but anxious patient with tachycardia, mild left lower quadrant tenderness without guarding or rigidity, and clear lungs. Vital signs were notable for a heart rate of 117 beats per minute, blood pressure of 133/77 mmHg, and a low-grade temperature of 99.2°F. *C. difficile* toxin A and B testing was ordered on day 107, yielding a positive result on day 109, confirming recurrent infection. Management required referral back to gastroenterology to discuss fetal microbiota transplantation, given her refractory disease course in the setting of chronic immunosuppression from hidradenitis suppurative therapy and underlying comorbidities.

There was overall improvement noted by her infectious disease specialist on day 134, with the patient reporting a decrease in bowel movement frequency from four to five episodes daily to two to three, approaching her baseline of one to two. The specialist was optimistic that disease clearance had been achieved with completion of her third round of fidaxomicin and recommended continued observation off antibiotics with close follow-up. The patient remained asymptomatic at her subsequent visit, with repeat *C. difficile* stool toxin testing reserved for recurrence of diarrhea. Follow-up laboratory testing on day 169 demonstrated improvement in renal function and resolution of acute electrolyte disturbances, though persistent hyperkalemia and metabolic acidosis remained. *C. difficile* stool toxin testing at this time returned negative (Table 1).

In spite of eight months of treatment and monthly follow-up visits with her primary care physician and two specialists, the patient reported an increase in bowel movements during a visit with her infectious disease specialist on day 199. Physical examination revealed a rash in the right axilla, diagnosed as impetigo likely secondary to a hidradenitis suppurativa flare. The patient's dermatologist prescribed mupirocin twice daily for 14 days, given the contraindication to antibiotic use. The absence of other accompanying symptoms, however, raised consideration of an alternative etiology. Her infectious disease specialist recommended continued observation off antibiotics, repeat stool testing, and close follow-up given the concern for disease recurrence. On day 234, the patient presented to her gastroenterologist, reporting loose stools for the preceding 14 days accompanied by bloating, back pain, dizziness, fatigue, and heartburn. *C. difficile* stool toxin A and B were ordered, and fecal microbiota transplantation was recommended. The patient's clinical course remained ongoing, with further management pending the results of repeat stool toxin testing and multidisciplinary discussion regarding fecal microbiota transplantation.

## Discussion

In recent years, infection with *C. difficile* has become one of the most detrimental healthcare-associated infections, with cases increasing in both frequency and severity. Although antibiotics are invaluable in modern medicine, they carry risks such as profound dysbiosis and the emergence of resistant strains. Elderly patients are disproportionately prone to complications once the gut flora is compromised by antibiotics [1]. The interaction between dermatologic treatment and gastrointestinal health is highlighted by this patient's clinical course. Although clindamycin is commonly used for several dermatologic conditions, it is among the highest-risk antibiotics for CDI due to severe disruption of protective anaerobic flora [4,7]. In this patient, chronic antibiotic use rather than acute exposure to clindamycin likely created persistent dysbiosis favoring *C. difficile* overgrowth. Despite administration of the current gold-standard therapies, fidaxomicin and vancomycin, infection can persist and enter a cycle of recurrence [8]. Long-term antibiotic use should be avoided unless absolutely necessary in order to avoid dysbiosis and the depletion of protective commensal microbiota.

The patient's failure to respond to fidaxomicin, which in randomized trials has demonstrated superior sustained clinical cure rates compared to vancomycin [2,9,10], suggests impaired mucosal healing beyond simple bacterial resistance. Anti-TNF therapy is also used in many dermatologic conditions like HS, and likely contributed significantly to the CDI treatment failure. TNF-alpha coordinates innate immune responses to CDI, facilitating neutrophil recruitment and maintaining epithelial barrier integrity [11]. By neutralizing TNF-alpha to control HS, the patient's intestinal defenses against *C. difficile* toxins were compromised. Research indicates that CDI recurrences occur more frequently in immunocompromised patients compared to the general population [12]. With all of these protective mechanisms blunted, conventional therapy with antibiotics was not able to restore homeostasis of the gut microbiota.

HS is increasingly recognized as a systemic inflammatory condition with significant gastrointestinal comorbidities. A bidirectional association between HS and inflammatory bowel disease (IBD), including Crohn's disease and ulcerative colitis, has been well documented, with emerging evidence supporting a causal effect of IBD on the development of HS [13]. Both conditions share common pathogenic mechanisms, including immune dysregulation, aberrant cytokine signaling, and dysbiosis. Importantly, differentiating perianal HS from perianal Crohn's disease presents a diagnostic challenge, particularly relevant in patients like ours with perianal involvement [13]. Clinicians managing HS patients on immunosuppressive therapy should routinely screen for gastrointestinal symptoms and refer to gastroenterology when indicated, as the presence of concurrent IBD may further predispose patients to opportunistic infections, including CDI.

There are nonpharmacological factors at play that could also be contributing to this patient’s CDI persistence. The patient’s comorbidities, such as CKD and T2DM, create a physiological environment that may have hindered normal recovery mechanisms. CKD leads to uremic toxin accumulation that alters gut microbiota composition and disrupts intestinal barrier function [14]. Without this barrier, the gut is more susceptible to inflammation and infection. T2DM also impairs immune function and neutrophil function, and increases the risk and severity of infections [15]. These factors likely prevented adequate mucosal repair and protection even when fidaxomicin reduced bacterial burden.

To maximize treatment efficacy and achieve potential eradication, studies have pivoted towards optimizing dosage schedules and delivery methods of existing therapies. Extended-pulsed fidaxomicin regimens have shown promise in elderly patients at high risk for recurrence [10], however, data for immunocompromised patients receiving concomitant antibiotics remain mixed [16]. While some trials demonstrate improved outcomes with fidaxomicin in specific immunocompromised populations, the unique combination of chronic clindamycin exposure, anti-TNF therapy, CKD, and diabetes may have created especially refractory conditions in this patient. A meta-analysis showed that while fidaxomicin reduced persistent diarrhea, recurrence, and death by 40% compared to vancomycin overall, certain high-risk subgroups may not achieve these benefits [17]. This emphasizes the need for more research on other treatment options.

In cases where standard antibiotic treatment is ineffective in curing the CDI, fecal microbiota transplantation is often considered to be the next course of action to restore a healthy gut microbiome. Data have shown that outcomes are different depending on the fecal delivery method; some delivery methods include colonoscopy, retention enema, oral capsules, and nasojejunal/nasoduodenal tubes. Fecal microbiota transplantation treatment has not yet been standardized; however, it is a highly promising treatment for refractory CDIs [1]. Another promising pharmacologic agent against refractory CDIs is bezlotoxumab, a monoclonal antibody against *C. difficile*toxin B. It has been indicated for use in high-risk patients to prevent CDI recurrence [18]. More research is needed on potential methods and combinations of therapies in order to standardize treatment for patients with recurrent CDI.

## Conclusions

This case highlights the challenges in managing recurrent CDI. Treatment failure with fidaxomicin in patients on anti-TNF therapy may reflect host immune deficiency rather than antibiotic resistance alone. The combination of clindamycin-induced dysbiosis, TNF-alpha inhibition, and uremia-driven microbiome alterations created persistent intestinal vulnerability. Long-term antibiotic use should be avoided unless absolutely necessary, especially for patients at a higher risk for CDI. 

For patients who fail to recover with fidaxomicin treatment while on biologic agents, clinicians should consider early interventions beyond the standard antibiotic regimen. Some other options include temporary suspension of biologic therapy if medically feasible, early fecal microbiota transplantation to restore microbiome diversity, and bezlotoxumab for passive immunity against *C. difficile* toxin B. Managing such cases requires balancing control of inflammatory dermatologic disease with preservation of gastrointestinal immune function. Even optimal antibiotic selection may fail to eradicate a CDI when underlying immunologic defenses are pharmacologically suppressed, as demonstrated in this patient.
